# Impact on healthcare resource utilization of multiple sclerosis in Spain

**DOI:** 10.1186/s12913-017-2807-x

**Published:** 2017-12-29

**Authors:** Antoni Sicras-Mainar, Elena Ruíz-Beato, Ruth Navarro-Artieda, Jorge Maurino

**Affiliations:** 1 Fundación Rediss (Red de Investigación en Servicios Sanitarios), Barcelona, Spain; 2 Health Economics and Outcomes Research Unit, Roche Farma, Madrid, Spain; 3 Department of Medical Information, Hospital Universitari Germans Trias i Pujol, Badalona, Barcelona Spain; 4 Medical Department, Roche Farma, Madrid, Spain; 5 Madrid, Spain

**Keywords:** Multiple sclerosis, Healthcare resource utilization, Costs, Electronic medical records

## Abstract

**Background:**

Multiple sclerosis (MS) is a chronic disease with a high socioeconomic impact. The aim of this study was to assess healthcare resources utilization and costs in a sample of patients with MS.

**Methods:**

A retrospective, cohort study was conducted using electronic medical records from 19 primary care centres in Asturias and Catalonia, Spain. Adult patients diagnosed with MS were distributed into two groups according to the Expanded Disability Status Scale (EDSS) score: 0–3.5 (no-moderate disability) and 4–9.5 (severe disability). Healthcare (direct cost) and non-healthcare costs (work productivity losses) were analysed. An analysis of covariance (ANCOVA) was used for correction, *p* < 0.05. A multiple regression model was performed to obtain the variables associated with costs.

**Results:**

A total of 222 patients were analyzed; mean (SD) age: 45.5 (12.5) years, 64.4% female, and 62.2% presented a diagnosis of relapsing-remitting MS. Median EDSS score was 2.5, with 68.5% of the patients with no to moderate disability. The mean annual cost per MS patient was €25,103. For no-moderate and severe disability, the ANCOVA-adjusted mean annual cost was €23,157 and €29,242, respectively (*p* = 0.013). Direct costs and MS disease-modifying therapy accounted for 39.4% and 31.7% of the total costs, respectively. The total costs were associated with number of relapses (β = 0.135, *p* = 0.001), time since diagnosis (β = 0.281, *p* = 0.023), and age (β = 0.198, *p* = 0.037).

**Conclusions:**

Multiple sclerosis imposes a substantial economic burden on the Spanish National Health System, patients and society as a whole. Costs significantly correlated with disease progression.

## Background

Multiple sclerosis (MS) is a chronic, autoimmune disease characterised by inflammation of the central nervous system that leads to demyelination, axonal loss and progressive neuronal degeneration [1]. A prevalence of 125 cases/100,000 inhabitants was found in Spain affecting mainly young adults [2–5].

Multiple sclerosis progresses from episodic attacks followed by periods of remission (relapsing-remitting MS [RRMS]) to a more progressive state (secondary progressive MS [SPMS]) in approximately 80% of patients [1]. Primary progressive MS (PPMS) accounts for 10% of the overall population with MS and differs from RRMS and SPMS patients, in that progression consists of gradual worsening of neurologic disability from symptom onset [1]. Disease progression is linked to the accumulation of disability, which overall, is faster for patients with PPMS than for patients with RRMS [1]. Current MS disease-modifying therapies (DMTs) are used with the aim of reducing the number of relapses, their severity, and slowing the disability’s progression [6].

The age of onset of MS is generally in the most financially productive time of the patients’ lives and consequently has a substantial economic burden on patients, their families, and society as a whole [7–10]. The European total annual cost of MS was €14.6 billion in 2010 (1.8% of the total annual economic cost of all brain disorders) [11]. A recent study involving 16,808 patients with MS in 16 European countries found that work capacity of MS patients declined from 82 to 8% with advancing disease, and utility declined from normal population values to less than zero [12]. Patients with PPMS present higher healthcare utilization than patients with SPMS and RRMS, due to different provider visits, emergency visits, and hospital admissions [13]. Disease-modifying therapies are the main cost drivers for patients with mild disease severity, while for those with more advanced disability these are production losses and informal care [10, 12].

Mental and neurological disorders also have a substantial economic impact in Spain (equivalent to almost 8% of the country’s GDP) with a mean yearly per-patient cost of €30,050 for MS patients [14, 15]. However, information about the use of healthcare resources and associated costs among patients with MS in Spain is limited. The objective of this study was to assess the healthcare resources utilization of MS patients in Spain according to the degree of disability in order to provide detailed and updated information about the economic burden of the disease.

## Methods

### Study design

This study was a secondary analysis of electronic health records from 19 primary care centres in two regions of Spain (Asturias and Catalonia). The investigational review board of the Fundació Unió Catalana d’Hospitals (Barcelona) approved the protocol.

### Study population

Key inclusion criteria were: age ≥ 18 years, a diagnosis of MS (International Classification of Primary Care and International Statistical Classification of Diseases, ninth revision criteria), requiring medical care from 2010 to 2015, and being in the long-term prescriptions program with a follow-up of ≥2 records in the computer system [16, 17].

### Disability assessment

The Expanded Disability Status Scale (EDSS) is a clinician-administered scale that is.

widely used in both clinical trials and routine clinical practice to assess the clinical severity and the functional impairment of MS [18]. The score is based on measures of impairment in eight functional systems: pyramidal, cerebellar, brainstem, sensory, bowel and bladder function, visual function, cognition, and ‘other’. Each functional system is scored on a scale of 0 (no disability) to 5 or 6 (severe disability). The overall EDSS score ranges from 0 to 10 with higher scores indicating increased levels of disability.

### Demographic and clinical variables

The following demographic and clinical variables were collected: age, gender, type of MS (RRMS, PPMS, SPMS and clinically isolated syndrome [CIS]), time since diagnosis, comorbidity and pharmacological treatments using the Anatomical Therapeutic Chemical Classification System (ATC) [19]. The number of chronic diseases, the Charlson Comorbidity Index, and Case-mix Index obtained from the Adjusted Clinical Groups (ACG) were used to summarize general comorbidity [20, 21].

### Healthcare resources and costs

Direct healthcare costs were those related to healthcare activity (medical visits, hospitalisation days, emergency visits, diagnostic or therapeutic procedures and medication) and indirect costs related to work productivity loss (days off work due to sick leave). The cost was expressed as mean cost per patient (annual mean). Healthcare resources (€, year 2014) are shown in Table 1 and are expressed as mean cost per patient (cost/unit). The unit costs were obtained directly from the study centres with the exception of medication costs and work productivity loss. Prescriptions (acute, chronic, or upon request) were quantified according to the recommended retail price per package at the time of prescription (Bot Plus database) [22]. The days absent from work were collected from a specific computer program managed by primary care physicians and quantified according to the official minimum wage salary (source: *Instituto Nacional de Estadística*-INE, Spanish National Statistics Institute) [23]. A sub-analysis of resource was performed in patients stratified by type of MS (RRMS vs. PPMS) using the above calculations.

**Table 1:** Use of healthcare resources and unit costs

	Unit costs (EUR)
Medical visits	
Primary care	23.19
Emergency care	117.53
Hospitalisation (one day)	320.90
Special care^a^	67.50
Additional tests	
Laboratory tests	22.30
Conventional radiology tests	18.50
Other diagnostic/therapeutic tests	47.12
Pharmaceutical prescription	Retail price/pack
Work productivity–indirect costs	
Cost per day of sick leave	79.4

Source of healthcare resources: own analytical accounts and INE. Values expressed in euros (year 2014). Retail price includes value-added tax. ^a^Neurology visits

### Statistical analysis

Demographic, clinical and economic variables were collected for the overall sample of valid patients and for stratified subgroups according to the EDSS score: no disability -moderate disability (0.0–3.5) and severe disability (4.0–9.5). A descriptive analysis was presented for all variables of interest with mean values, standard deviation (SD) and 95% confidence intervals (CIs). Normal data distribution was verified using a Kolmogorov-Smirnov test. Costs were compared by analysis of covariance (ANCOVA) of age, gender, RUBs, Charlson Comorbidity Index, and time since MS diagnosis (generalized linear model). The bivariate analysis included ANOVA, the chi-squared test, Pearson’s linear correlation, and comparison of means. A multiple linear regression model was used to evaluate the variables associated to the costs (stepwise method) including age, gender, RUBs, Charlson Comorbidity Index, time since MS diagnosis, and EDSS score. The Statistical Package for the Social Sciences-Windows (SPSSWIN) version 19 was used. A *p*-value < 0.05 was considered to be statistically significant.

## Results

**Fig. 1:**
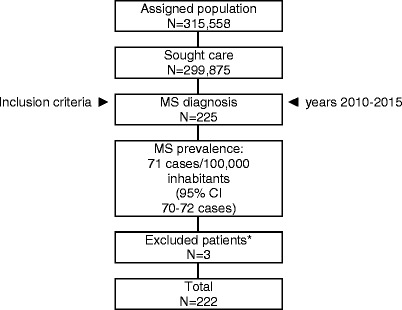
Study disposition. *Patients were excluded due to missing or inconsistent data (*n* = 2) and loss of follow-up (*n* = 1). MS: Multiple Sclerosis

A total of 222 patients were included (Figure 1). The mean (SD) age was 45.5 (12.5) years and 64.4% were female. Prevalence of MS was 71 cases/100,000 inhabitants. Relapsing-remitting MS was the most common clinical form (62.2% of the patients). The median EDSS score was 2.5 (range: 1.0–8.5). The impact of comorbidity was significantly greater in the severe disability group vs. the no to moderate disability group: mean number of comorbidities (6.0 vs. 4.5; *p* = 0.001), Charlson Index (1.0 vs. 0.7; *p* = 0.005), and RUBs (3.2 vs. 2.9; *p* = 0.003). Demographic and clinical characteristics of the patients are shown in Table 2. Intramuscular interferon beta-1a (30.6%), subcutaneous interferon beta-1a (23.9%) and glatiramer acetate (18%) were the most common disease-modifying treatments administered.

**Table 2:** Demographic and clinical characteristics

	EDSS 0–3.5	EDSS 4.0–9.5	Total	*p*-value
	*N* = 152	*N* = 70	*N* = 222	
Geographic regions in Spain, %				
Asturias	30.9	22.9	28.4	0,587
Catalonia	69.1	77.1	71.6	0,827
Age, years, mean (SD)	42.5 (11.5)	52.2 (12.2)	45.5 (12.5)	<0.001
Gender, female, N (%)	68.4	65.7	64.4	0.292
Time since diagnostic, years, mean (SD)	10.5 (7.6)	19.8 (10.2)	13.4 (9.5)	<0.001
MS type, %				
RRMS	74.3	35.7	62.2	<0.001
SPMS	13.8	50.0	25.2	<0.001
PPMS	7.2	14.3	9.5	0.009
CIS	4.6	0.0	3.2	<0.001
Relapses during follow-up				
Number of relapses, mean (SD)	0.4 (0.6)	1.2 (1.0)	0.7 (0.9)	<0.001
Proportion of population, N (%)				
≥ 1	36.8	67.1	46.4	<0.001
1	29.6	18.6	26.1	0.008
2	6.6	40.0	17.1	<0.001
3	0.7	8.6	3.2	0.790
Comorbidity, mean (SD)				
Number of comorbidities	4.5 (2.7)	6.0 (3.3)	5.0 (3.0)	0.001
Charlson Index	0.7 (0.6)	1.0 (0.7)	0.8 (0.6)	0.005
RUBs, mean	2.9 (0.8)	3.2 (0.7)	3.0 (0.8)	0.003
Proportion of population, N (%)				
1 (healthy o very low morbidity)	3.3	0.0	2.3	<0.001
2 (low morbidity)	25.7	14.3	22.1	0.038
3 (moderate morbidity)	52.0	54.3	52.7	0.873
4 (high morbidity)	18.4	28.6	21.6	0.041
5 (very high morbidity)	0.7	2.9	1.4	0.031

*CIS* clinically isolated syndrome, *EDSS* expanded disability status scale, *MS* multiple sclerosis, *PPMS* primary progressive MS, *RRMS* relapsing remitting MS, *RUBs* resource utilization bands, *SD* standard deviation, *SPMS* secondary-progressive MS

### Healthcare resource use

Table 3 shows the mean annual use of healthcare resources by each disability group. Patients in the severe disability group, presented a significantly higher number of primary care medical visits (12.3 vs. 8.5; *p* < 0.001), specialised medical visits (3.0 vs. 2.3; *p* = 0.008), emergency room visits (2.0 vs. 1.2; p < 0.001), and hospitalisation days (5.6 vs. 1.3 days; p < 0.001) compared to patients in the no-moderate disability group. Mean (SD) work productivity losses were also significantly higher for the severe disability group vs. to the no-moderate disability group: 258.1 (162.0) vs. 160.9 (173.4) days, *p* < 0.001, respectively.

**Table 3:** Healthcare resource use and costs

	EDSS 0–3.5	EDSS 4.0–9.5	Total	*p*-value
	*N* = 152	*N* = 70	*N* = 222	
Annual number per patient, mean (SD)				
Medical visits, primary care	8.5 (5.7)	12.3 (5.7)	9.7 (6.0)	<0.001
Medical visits, specialists	2.3 (1.5)	3.0 (2.0)	2.5 (1.7)	0.008
Laboratory tests	2.8 (2.6)	3.7 (2.7)	3.1 (2.7)	0.010
Radiology tests	1.9 (2.5)	3.2 (2.6)	2.3 (2.6)	<0.001
Additional tests	1.6 (2.3)	3.4 (3.8)	2.2 (3.0)	<0.001
Hospitalisation days	1.3 (3.8)	5.6 (6.7)	2.7 (5.3)	<0.001
ER visits	1.2 (1.3)	2.0 (1.5)	1.5 (1.4)	<0.001
Work productivity losses, days	160.9 (173.4)	258.1 (162.0)	191.6 (175.5)	<0.001

*EDSS* Expanded Disability Status Scale, *ER* emergency room, *SD* standard deviation

Patients with PPMS showed a significantly higher frequency of additional tests use (other than laboratory and radiology tests), days of hospitalisations (both *p* ≤ 0.001), radiological tests use (*p* = 0.001), and specialised medical visits (*p* = 0.012) compared to RRMS patients (Table 4).

**Table 4:** Healthcare resource use and costs according to MS type

	RRMS	PPMS	Total	*p*-value
	*N* = 152	*N* = 70	*N* = 222	
Annual number per patient, mean (SD)				
Medical visits, primary care	8.9 (5.9)	10.0 (7.1)	9.1 (6.0)	0.426
Medical visits, specialists	2.3 (1.6)	3.3 (1.7)	2.4 (1.7)	0.012
Laboratory tests	2.9 (2.8)	3.3 (1.8)	3.0 (2.7)	0.589
Radiology tests	1.6 (2.0)	3.3 (2.2)	1.8 (2.1)	0.001
Additional tests	1.7 (2.6)	4.6 (4.1)	2.1 (3.0)	<0.001
Hospitalisation days	1.4 (4.0)	5.4 (6.3)	1.9 (4.6)	<0.001
Hospital emergencies	1.3 (1.3)	1.7 (1.8)	1.4 (1.4)	0.237
Work productivity losses, days	177 (174.1)	123.3 (175.2)	169.9 (174.7)	0.190

*MS* multiple sclerosis, *PPMS* primary progressive MS, *RRMS* relapsing remitting MS, *SD* standard deviation

### Direct healthcare costs and indirect costs

Table 5 specifies the direct healthcare costs and indirect costs (unadjusted and adjusted values) by disability group. From the total costs, 34.7% were related to primary care and 4.8% to specialised care. Disease-modifying therapies accounted for 31.7% of the total cost.

**Table 5:** Direct healthcare and indirect annual costs per MS patient (in EUR) according to disability level

	EDSS 0–3.5	EDSS 4.0–9.5	Total	*P*-value
	*N* = 152	*N* = 70	*N* = 222	
Unadjusted costs				
Annual cost (€) per patient, mean (SD)				
Direct healthcare costs	9331.7 (5504.5)	11,112.2 (6649.9)	9893.2 (5932.7)	0.037
Primary care	8614.7 (5199.4)	8887.5 (6178.4)	8700.7 (5514.1)	0.733
Medical visits	197.0 (132.9)	285.6 (131.6)	224.9 (138.5)	<0.001
Lab tests	61.3 (59.0)	83.5 (59.6)	68.3 (60.0)	0.010
Conventional radiology	34.6 (45.6)	59.7 (48.0)	42.5 (47.8)	<0.001
Supplementary tests	75.3 (110.5)	158.2 (181.4)	101.5 (141.8)	<0.001
Medication	272.8 (173.4)	417.6 (193.1)	318.5 (191.6)	<0.001
MS-specific drugs	7973.7 (5196.7)	7882.9 (6186.7)	7945.0 (5514.0)	0.910
Specialised care	717.0 (1361.2)	2224.7 (2342.7)	1192.5 (1863.5)	<0.001
Hospitalisations	418.0 (1211.5)	1792.5 (2147.8)	851.4 (1689.0)	<0.001
Medical visits	156.8 (102.4)	200.6 (134.0)	170.6 (114.8)	0.008
Emergency room visits	142.3 (156.9)	231.7 (180.1)	170.5 (169.4)	<0.001
Indirect costs^a^	12,775.0 (13,768.9)	20,495.4 (12,862.0)	15,209.4 (13,932.3)	<0.001
Total costs	22,106.8 (15,313.8)	31,607.6 (14,435.0)	25,102.5 (15,648.1)	<0.001
Adjusted costs^b^			Difference between unadjusted and adjusted costs	
Annual cost (€) per patient, mean (95% CI)				
Direct Healthcare costs	9124 (8100, 10,147)	11,476 (9988, 12,963)	2352	0.015
Primary care	8331 (7364, 9296)	9477 (8072, 10,881)	1146	0.205
Specialised care	793 (499, 1087)	1999 (1571, 2426)	1206	<0.001
Indirect costs (productivity)	14,033 (11,715, 16,351)	17,766 (14,395, 21,136)	3733	0.049
Total costs	23,157 (20,561, 25,753)	29,242 (25,467, 33,016)	6085	0.013

*95% CI* 95% confidence interval, *EDSS* expanded disability status scale, *SD* standard deviation. ^a^Indirect costs: loss of work productivity. ^b^ANCOVA model: costs adjusted by covariables (age, gender, resource utilization bands [RUBs], Charlson comorbidity index, and time since diagnosis of the multiple sclerosis)

The total cost for the 222 study patients was 5.6 million euros, 39.4% of which were direct healthcare costs. The total annual mean cost (direct and indirect) per MS patient was €25,103. This mean cost was significantly higher in the severe disability (vs. no-moderate disability) group (€31,608 vs. €22,107 *p* < 0.001), predominantly due to a higher healthcare resource utilization, in particular primary care visits (12.3 vs. 8.5; p < 0.001).

In the adjusted model (ANCOVA) this mean (95% CI) difference in total cost was maintained: severe disability €29,242 (€25,467, €33,016) vs. no-moderate disability €23,157 (€20,561, €25,753); a statistically significant difference of approximately €6085 was observed (*p* = 0.013). In the multiple regression model, the total costs were associated to the number of relapses (β = 0.135, 95% CI 10.2–105.1, *p* = 0.001), time since diagnosis (β = 0.281, 95% CI -272.2-11.8, *p* = 0.023) and age (β = 0.198, 95% CI 3.1–195.2, *p* = 0.037). The EDSS score was included in the model but was not significant. The model’s coefficient of determination was 33.5%. There were no significant differences between the evaluated variables by geographical regions. It is worth noting that 50.5% of the patients in the whole sample were unemployed due to their disability.

## Discussion

Multiple sclerosis is a chronic disabling disease that is associated with reduced quality of life and a high socioeconomic impact [7]. Physical disability at diagnosis is the main determinant of the economic burden, with 13% increased annual costs for each additional point from baseline EDSS [24]. In addition, the costs increase with more severe disability, especially when patients lose their upper limb function and independence (EDDS score > 7.0) [25].

The therapeutic landscape of treatment has changed dramatically over the last years. An increasing number of new drugs have recently shown encouraging results for the management of RRMS due to their proven higher efficacies compared to first-generation DMTs [6]. However, despite the availability of more treatment options, costs for all DMTs have increased substantially [10]. In addition, the early disease onset of MS has a significant impact on the patient’s most productive working years, leading to huge potential societal costs associated with this productivity loss [10]. Patients are less likely to be employed, are more likely to require time off work and to retire early compared to people without MS [7, 9, 12].

The impact on the costs of managing patients with MS is increasingly an area of interest [26–29]. However, comparing costs between countries with different socioeconomic, cultural, epidemiological background, and different systems for organizing and funding healthcare is very difficult [12, 30].

This study shows an overall annual mean cost per MS patient of €25,103. This mean cost was significantly higher in patients with severe disability compared than those with no-moderate disability (€29,242 and €23,157, respectively; *p* = 0.013). Total cost was associated to the number of relapses, time since diagnosis, and age (*p* = 0.001, 0.023, and 0.037, respectively). Indirect costs and MS therapy accounted for 61% and 31.7% of the total costs, respectively.

In Spain, the available data for cost of MS is limited [12, 31–34]. These findings concur with prior studies. In a cost-of-illness analysis based on information from 1848 patients, Kobelt et al. found that the total mean costs per patient were driven by the distribution of the disease severity levels [30]. Workforce participation decreased from approximately 70% in the early disease stages to less than 5% in the very late stages. Productivity losses increased more than eightfold in patients with an EDSS score of 0–1 vs. 8–9. In another study with a sample of 200 MS patients in Barcelona, Casado et al. found that the main drivers for direct costs were DMTs in low disability stages and caregiver costs in severe disability stages [32]. Overall, direct healthcare costs accounted for 60% of total cost; within these direct costs DMTs accounted for 78% in the early disability stages to 11% in the later disability stages. The correlation of disability with the increasing economic burden of MS was also shown in the TRIBUNE study [34]. The mean cost per patient per year was €20,659 for patients with mild disease severity, while patients with moderate MS incurred more than double that cost (€43,948). DMTs were the most expensive cost component for patients with mild and moderate disability (58% and 32%, respectively) [34].

Missing values and differences on diagnostic codification are usual limitations related to studies with population databases [35]. McDonald 2010 criteria were not used in the study because our healthcare database collected diagnosis following only IPC-2 and ICD-9 classifications. In addition, concomitant medications were not evaluated. This study did not include any non-healthcare direct costs, classified as “out-of-pocket” costs paid by the patient/family, as they were not recorded in the database. The only direct costs considered were those relating to the public health system and the area of influence of the patient. Another limitation was the absence of informal caregivers to calculate informal costs. Sick leave (temporary or permanent) may in turn be a limited indicator of indirect costs as premature death and informal costs were not considered. In addition, standard cost for sick leave should have been applied, rather than the specific costs depending on patients’ income. Despite these limitations, these results reflect the economic impact of MS and how these vary between different disability levels.

## Conclusions

Patients with MS show high healthcare resource utilization and large work productivity losses that cumulatively impose a substantial economic burden on the healthcare system and society as a whole. This burden was enhanced upon disease progression.

Therefore, a more proactive management strategy, including earlier use of high-efficacy DMTs and close monitoring of the clinical and radiological response to treatment, is recommended to slow or halt the progression of physical and cognitive impairments in patients with MS [36, 37]. No evidence of disease activity is emerging as a new standard MS outcome and may be associated with improved long-term disability.

Additional research focusing on direct healthcare and indirect costs as well as standardised methodologies to calculate costs are necessary to determine the association between the disease evolution and economic burden.

## Abbreviations

ACG: Adjusted clinical groups;
CIS: Clinically isolated syndrome
EDSS: Expanded disability status scale
ICD-9: International statistical classification of diseases
IPC-2: International classification of primary care
MS: Multiple sclerosis
PPMS: Primary progressive multiple sclerosis
RRMS: Relapsing-remitting multiple sclerosis
RUBs: Resource utilization bands
SD: Standard deviation
SPMS: Secondary progressive multiple sclerosis
